# Ethanol contamination of cerebrospinal fluid during standardized sampling and its effect on ^1^H-NMR metabolomics

**DOI:** 10.1007/s00216-015-8663-9

**Published:** 2015-05-03

**Authors:** Sonia A. van der Sar, Ronald Zielman, Gisela M. Terwindt, Arn M. J. M. van den Maagdenberg, André M. Deelder, Oleg A. Mayboroda, Axel Meissner, Michel D. Ferrari

**Affiliations:** Center for Proteomics and Metabolomics, Leiden University Medical Center, Albinusdreef 2, 2333 ZA Leiden, The Netherlands; Department of Neurology, Leiden University Medical Center, P.O. Box 9600, 2300 WB Leiden, The Netherlands; Department of Human Genetics, Leiden University Medical Center, P.O. Box 9600, 2300 RC Leiden, The Netherlands

**Keywords:** Metabolomics, Cerebrospinal fluid, Ethanol, NMR, Biobank

## Abstract

Standardization of body fluid sampling, processing and storage procedures is pivotal to ensure data quality in metabolomics studies. Yet, despite strict adherence to standard sampling guidelines, we detected variable levels of ethanol in the ^1^H-NMR spectra of human cerebrospinal fluid (CSF) samples (range 9.2 × 10^−3^–10.0 mM). The presence of ethanol in all samples and the wide range of concentrations clearly indicated contamination of the samples of some sort, which affected the ^1^H-NMR spectra quality and the interpretation. To determine where in the sampling protocol the ethanol contamination occurs, we performed a CSF sampling protocol simulation with 0.9 % NaCl (saline) instead of CSF and detected ethanol in all simulation samples. Ethanol diffusion through air during sampling and preparation stages appeared the only logical explanation. With a bench study, we showed that ethanol easily diffuses into ex vivo CSF samples via air transmission. Ethanol originated from routinely used skin disinfectants containing ethanol and from laboratory procedures. Ethanol affected the CSF sample matrix at concentrations above ~9.4 mM and obscured a significant part of the ^1^H-NMR spectrum. CSF sample preparation for ^1^H-NMR-based metabolomics analyses should therefore be carried out in a well-ventilated atmosphere with laminar flow, and use of ethanol should be avoided.

**Electronic supplementary material:**

The online version of this article (doi:10.1007/s00216-015-8663-9) contains supplementary material, which is available to authorized users.

## Introduction

Cerebrospinal fluid (CSF) is generally believed to reflect brain physiology and is therefore frequently used to assess biomarkers for brain disorders [1–3]. CSF sampling, processing, and storage procedures are pivotal to prevent biochemical ex vivo changes which may invalidate the observed profiles. Recently, a consensus protocol was published [4] to standardize CSF sampling and storage for CSF biobanks. However, despite rigorous adherence to this and other protocols, we detected substantial amounts of ethanol disturbing the ^1^H-NMR spectra of human CSF samples. Other studies reporting the presence of ethanol in CSF have interpreted this as either a contaminant [5, 6] or due to the disease process [7, 8].

In the present study, we set out to identify the origin of ethanol contamination and to assess the effect it has on the sample matrix.

## Methods

### Human CSF samples

**Fig. 1:**
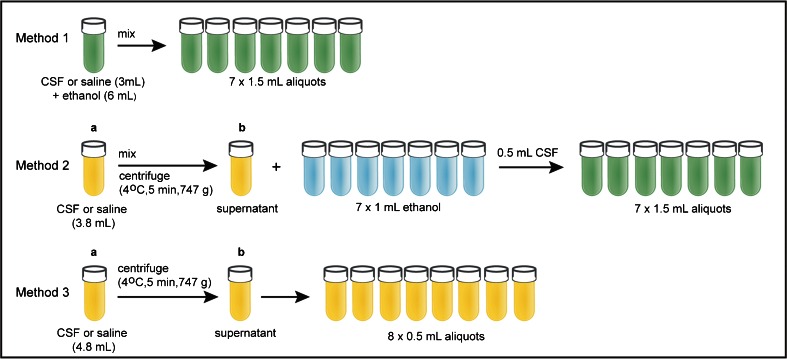
In-house sample processing protocol used for preparation of samples for mass spectrometry (MS)-based metabolomics (methods 1 and 2) and for ^1^H-NMR-based metabolomics (method 3). All aliquots were immediately placed on dry ice within 30 min of sampling and then transferred to −80 °C for storage within 60 min

The cohort in which we initially discovered ethanol contamination consisted of human CSF samples that were collected for research purposes, namely to investigate the pathophysiology of migraine. The study protocol was approved by the Medical Ethical Committee of Leiden University Medical Center (LUMC), and all subjects gave written informed consent prior to collection. For disinfection of the skin, chlorhexidine (5 g/L)/denatured ethanol 70 % (Pharmacy LUMC, art. no.: 909602) was used. CSF samples were taken via lumbar puncture. Three different sample handling protocols were used simultaneously for the preparation of samples for mass spectrometry (MS)-based metabolomics (methods 1 and 2) and for ^1^H-NMR-based metabolomics (method 3) (see Fig. 1). Method 3 was used for the initial ^1^H-NMR measurements in which we detected ethanol contamination. For sampling of method 3, 4.8 mL of CSF dripped through air into a 15-mL polypropylene falcon tube. Directly after sampling, the CSF was centrifuged at 4 °C for 5 min (2000 rpm, 747 g). Following centrifugation, the supernatant was transferred to a 15-mL polypropylene falcon tube and divided in 0.5-mL aliquots, placed on dry ice within 30 min of sampling and transferred to −80 °C for storage within 60 min of sampling. In sampling methods 1 and 2, cold ethanol was added to the CSF during the sample handling to denature proteins and to be able to thaw the samples before measurements at lower temperatures. Because this was a likely source of ethanol contamination, we also analysed three CSF samples obtained for clinical diagnostic purposes to check whether ethanol is also present in these samples. Disinfection of the skin with chlorhexidine (5 g/L)/denatured ethanol 70 %, sample collection and sample processing of these clinical samples were similar to the research samples with the exception that ethanol was not used anywhere in the handling of these samples. For additional details on sampling and processing of the research and clinical CSF samples, see the Electronic Supplementary Material (ESM).

### Simulation of CSF sampling protocol with saline

To determine the origin of ethanol during our sample processing procedure, we performed a CSF sampling protocol simulation. A 0.9 % NaCl solution (saline) was used instead of CSF in an exact simulation of the CSF sampling protocol (see Fig. 1). Different pipetting methods were used for mass spectrometry (MS)-based metabolomics (methods 1 and 2) and for ^1^H-NMR-based metabolomics (method 3). In methods 1 and 2, one pipette was used throughout the entire sampling protocol, including pipetting of ethanol. In method 3, a different pipette was used which had never been exposed to ethanol; only these samples were measured with ^1^H-NMR as described below.

### Air to sample diffusion of ethanol

**Fig. 2:**
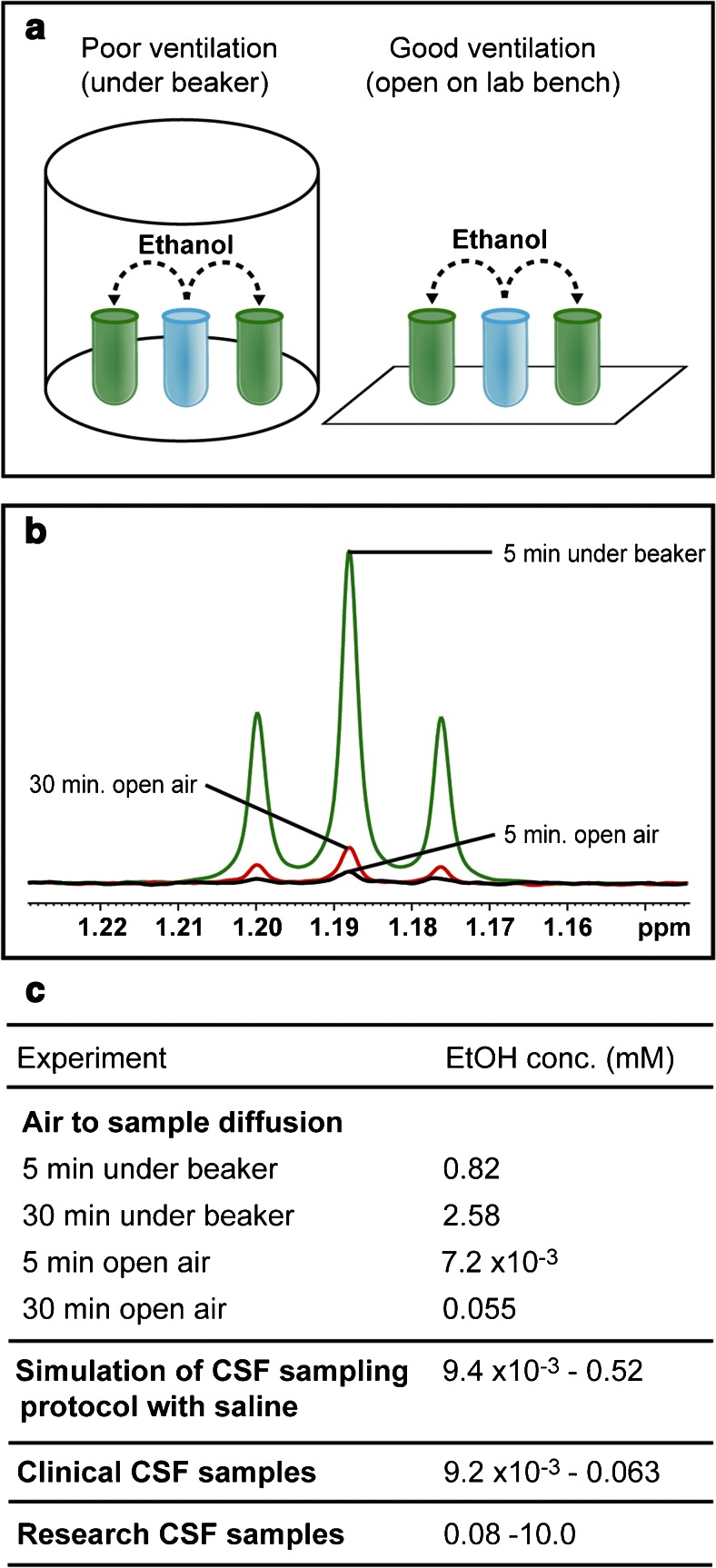
Cross-contamination of CSF by diffusion of ethanol through air. **a** Schematic representation of experimental set-up to test for air diffusion of ethanol. **b** ^1^H-NMR overlay spectrum showing the methyl signal of ethanol; here, it is used to visualize the relative amount of ethanol found in test samples from the air diffusion experiment. **c** Table comparing the amount of ethanol quantified in the air to sample diffusion test with the simulation of CSF sampling protocol with saline, clinical CSF samples and research CSF samples

To test how fast ethanol can diffuse into CSF via air, we created a work area simulation. Ethanol (1 mL, Biosolve, 96 % analytical grade) was placed into 2 × 1.8-mL cryotubes (Nunc, art. no. 368632). An up-turned 500-mL glass beaker was placed over two open cryotubes containing 1 mL of saline and one open cryotube containing ethanol (see Fig. 2a). After 5 min, one saline cryotube was removed and capped firmly until ^1^H-NMR analysis. The second cryotube was removed after 30 min. This process was repeated without the beaker in the same well-ventilated work space. All samples were then prepared for and analysed by quantitative ^1^H-NMR spectroscopy as described below.

### Effect of ethanol on the sample matrix

A serial dilution was made to test at which level of ethanol contamination the CSF matrix and thereby the whole ^1^H-NMR spectrum is affected. Two hundred microlitres of CSF was aliquoted into 10 × 1.5-mL eppendorf tubes containing ethanol (Biosolve, 96 % analytical grade), decreasing in concentration in regular stepped increments so that the final ethanol concentrations were 9.1 × 10^−3^–500.0 mM. Samples were prepared for ^1^H-NMR analysis as described below.

### ^1^H-NMR sample preparation

In 1.5-mL eppendorf tubes that were placed on ice, 225 μL of CSF, 0.9 % NaCl, or CSF/ethanol was added to 25 μL of pH 7.0 phosphate buffer (50 mM) in D2O containing 4 mM of sodium 3-trimethylsilyltetradeuteriopropionate (TSP) and 2.0 mM NaN_3_. Following thorough mixing by repeated inversion, 190 μL of the sample was transferred into 3-mm NMR tubes in a cooled rack at 6 °C.

### ^1^H-NMR data acquisition and processing

^1^H-NMR data were obtained using a Bruker 600 MHz AVANCE II spectrometer equipped with a 5-mm TCI cryoprobe and a z-gradient system; a Bruker SampleJet sample changer system was used for sample transfer; samples were kept at 6 °C while queued for acquisition. One-dimensional (1D) ^1^H-NMR spectra were recorded at 300.0 K using the first increment of a NOESY pulse sequence [9] with presaturation (γB1 = 50 Hz) during a relaxation delay of 4 s and a mixing time of 10 ms for efficient water suppression [10]. The duration of 90° pulses was automatically calibrated for each individual sample using a homonuclear gated nutation experiment [11] on the locked and shimmed samples after automatic tuning and matching of the probe head. Sixteen scans of 200,704 points covering 18,028 Hz were recorded and zero filled to 262,144 complex points prior to Fourier transformation; an exponential window function was applied with a line-broadening factor of 1.0 Hz. The spectra were manually phased and baseline corrected and automatically referenced to the internal standard (TSP = 0.0 ppm). Phase offset artifacts of the residual water resonance were manually corrected using a polynomial of degree 5 least square fit filtering of the free induction decay (FID) [12].

### ^1^H-NMR data analysis

Quantification of ethanol peaks was carried out by iterative line fitting in the frequency domain (Topspin version 2.1, Bruker Biospin; MDCON command).

## Results

### Ethanol in human CSF samples

Ethanol was detected in ^1^H-NMR spectra of not only all research CSF samples but also in three clinical CSF samples where ethanol is only used during disinfection and not during sample processing (see Fig. 2c). In the research samples, the ethanol levels varied between 0.08 and 10.0 mM and, in the three clinical samples, the ethanol levels were 9.2 × 10^−3^, 0.023 and 0.063 mM.

### Simulation of CSF sampling protocol with saline

In the simulation experiment, ethanol was detected in all samples, even in method 3 (Fig. 1) in which no ethanol was used. Furthermore, we observed a trend of increasing ethanol concentration from 0.070 to 0.203 mM (mean) in the aliquots of method 3, which was related to the time the cryotubes were uncapped during aliquoting. These findings suggested that the ethanol arose from cross-contamination by air diffusion during sampling and processing procedures.

### Air to sample diffusion of ethanol

To test the hypothesis of ethanol diffusion through air, we created a work area simulation. Ethanol readily diffused into an open vessel containing saline. After 5 min under a beaker, ethanol was detected at 0.82 mM compared to 7.2 × 10^−3^ mM after 5 min open on the lab bench. After 30 min, ethanol had increased to 2.5 and 0.055 mM, respectively (see Fig. 2b, c). These levels are in the same range as the CSF and saline samples prepared using sampling handling method 3 (see Fig. 2c).

### Effect of ethanol on the sample matrix

**Fig. 3:**
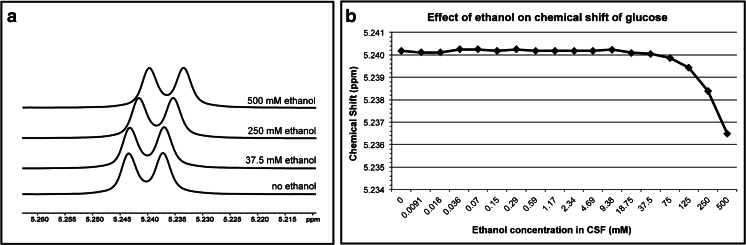
^1^H-NMR spectra overlay (**a**) and graph (**b**) showing the effect of ethanol on the chemical shift of the α-anomeric proton of d-glucose as an example

An effect of high ethanol concentrations on the CSF matrix was demonstrated in a spiking experiment. A distinctive upfield shift of small metabolite signals is observed at ethanol concentrations of 9.38 mM and higher (Fig. 3).

## Discussion

We showed that CSF samples can be contaminated with ethanol during routine sampling and processing steps via diffusion through air. Most likely sources of ethanol are (i) disinfection of the skin with chlorhexidine/70 % ethanol prior to lumbar puncture and (ii) use of ethanol in the same room as where the CSF samples were prepared for ^1^H-NMR measurements. Ethanol affected the CSF sample matrix at concentrations above ~9.4 mM and obscured a significant part of the ^1^H-NMR spectrum.

The most important effect of ethanol contamination is on the interpretation of the ^1^H-NMR spectra. The two signal groups of ethanol at 1.18 and 3.66 ppm can mask metabolites that lie in the same chemical shift region of ^1^H-NMR spectra. The methylene quartet at 3.66 ppm will itself be masked, as it lies in the heavily crowded glucose region of the spectrum. The methyl signal of the small metabolite 3-hydroxybutyric acid, however, normally occurs in the same region of the CSF spectrum as the ethanol methyl triplet (1.18 ppm). In the presence of ethanol, the detection and subsequent quantification of this metabolite are heavily compromised.

There are several solutions to minimize the risk of ethanol contamination and to mitigate its effects on metabolomics analysis, as follows: 1) ethanol regions can sometimes be removed from ^1^H-NMR spectra prior to multivariate analysis; 2) spectrum alignment might be required prior to analysis in cases of severe matrix distortion; 3) processing of CSF samples for ^1^H-NMR analysis in an ethanol-free zone or well-ventilated area such as a laminar flow cabinet; 4) use of non-ethanol-containing disinfectant instead of chlorhexidine/ethanol.

We believe that the recommendations resulting from this study should be considered when collecting CSF for biomarker research in brain disorders.

## Electronic supplementary material

ESM 1 (PDF 18 kb)
