# Supplementary material for: Ethanol contamination of cerebrospinal fluid during standardized sampling and its effect on 1H-NMR metabolomics
Source: Anal Bioanal Chem. 2015 May 3;407(16):4835–9. doi: 10.1007/s00216-015-8663-9 (PMC4446525; doi:10.1007/s00216-015-8663-9)
Supplement: Supplementary file 1 — (PDF 18 kb) [file 216_2015_8663_MOESM1_ESM.pdf]

Analytical and Bioanalytical Chemistry

Electronic Supplementary Material

Ethanol contamination of cerebrospinal fluid during standardized sampling and its effect on ^{1}H-NMR metabolomics

Sonia A. van der Sar, Ronald Zielman, Gisela M. Terwindt, Arn M.J.M. van den Maagdenberg, André M. Deelder, Oleg A. Mayboroda, Axel Meissner, Michel D. Ferrari

Sampling protocol for research CSF samples

Research CSF sampling was performed in the morning (before 12.00 a.m.) via lumbar puncture. All subjects were subjected to overnight fasting from at least 8 hrs before the lumbar puncture and were only allowed water prior to the lumbar puncture. Subjects underwent a neurological examination prior to the lumbar puncture to check for contra-indications for lumbar puncture. The procedures were performed by six, highly experienced Resident doctors in Neurology. Subjects were positioned in the left lateral recumbent knees-to-chest position. For disinfection of the skin Chlorhexidine (5g/L)/denatured ethanol 70% (Pharmacy LUMC, art. no. 909602) was used. CSF samples were taken via lumbar puncture between the L3/L4 or L4/L5 interspace with a Medi Plast 0.9(20G) x 90-mm traumatic needle. After penetrating the subarachnoid space the intracranial pressure was measured with a CSF manometer (Medisize B.V., art. no GDO02*S-A). For routine CSF diagnostics 3 mL CSF was sampled in a 12-mL polystyrene tube (Greiner, art. no. 160172).

Three different sample handling protocols were used for preparation of samples for MS-based metabolomics (methods 1 and 2) as well as for NMR-based metabolomics (method 3). All research samples were handled exactly the same way after sampling and all sample handling steps were carried out on ice.

For sampling method 1, 6 mL of cold ethanol (J.T.Baker, ethanol absolute, prod. no. 8098) was added to a 15-mL polypropylene falcon tube (Greiner, art.no. 188271) and placed on ice. Subsequently, 3.0 mL of CSF was sampled directly into this tube and inverted several times to mix thoroughly with the ethanol. Directly after sampling the CSF/Ethanol mixture was divided in 1.5-mL aliquots into 7 x 1.8-mL labeled cryotubes (Nunc 1.8-ml cryotubes – art. no. 368632), and placed on dry ice.

For sampling method 2, 3.8 mL of CSF was sampled in a 15-mL polypropylene falcon tube and centrifuged at 4°C for 5 minutes (2000 rpm, 747 g) directly after sampling. Following centrifugation, the supernatant from this tube was transferred to a 15-mL polypropylene falcon tube, and divided in 0.5-mL aliquots, into 7 x 1.8-mL cyrotubes (Nunc, 1.8-ml cryotubes – art.no. 368632) already containing 1 mL of cold ethanol. The aliquots were

repeatedly inverted to mix thoroughly and placed on dry ice.

For sampling method 3, 4.8 mL of CSF was sampled directly in a 15-mL polypropylene falcon tube and centrifuged at 4°C for 5 minutes (2000 rpm, 747 g) directly after sampling. Following centrifugation, the supernatant was transferred to a 15-mL polypropylene falcon tube, then divided in 0.5-mL aliquots into 8 x 1.0-mL cryotubes (Nunc 1.0-mL cryotubes – art. no. 366656), and placed on dry ice.

All sample aliquots were placed on dry ice within 30 minutes from sampling and were transferred to -80°C for storage within 60 minutes from sampling. All samples remained at -80 °C until sample preparation, no extra freeze-thaw cycles were allowed.

Sampling protocol for clinical CSF samples

Clinical CSF samples were collected at the Neurology outpatient clinic at the Leiden University Medical Center via lumbar puncture. The procedures were performed by three well-experienced residents in Neurology. For disinfection of the skin Chlorhexidine (5g/L)/denatured ethanol 70% (Pharmacy LUMC, art. no. 909602) was used. CSF samples were taken via lumbar puncture between the L3/L4 or L4/L5 interspace with a Medi Plast 0.9(20G) x 90-mm or 0.7(22G) x 70-mm traumatic needle. CSF was collected for routine CSF diagnostics and was sampled in a 12-mL polystyrene tube (Greiner, art. no. 160172). CSF was processed within 30 minutes after CSF collection. A small portion of CSF was used for cell count, and the remainder centrifuged at 21°C for 5 minutes (2000 rpm, 500 g). The supernatant was placed in a polyethylene tube (Roche, Twintube, art. no. 12029774001) and used for routine CSF measurements. The remaining CSF was stored in 2.0-ml polyethylene cryotubes (Sarstedt, Micro tube, art. no. 72.609), capped with a polyethylene screwcap (Sarstedt, Screw cap, green, art. no. 65.716.005), and transferred to a -20 °C freezer within 60 minutes from sampling. All clinical samples remained at -20 °C until sample preparation, no extra freeze-thaw cycles were allowed.
